# Prostate cancers with distinct transcriptional programs in Black and White men

**DOI:** 10.1186/s13073-024-01361-0

**Published:** 2024-07-23

**Authors:** Minhyung Kim, Patrick Tamukong, Gloria Cecilia Galvan, Qian Yang, Amanda De Hoedt, Michael R. Freeman, Sungyong You, Stephen Freedland

**Affiliations:** 1 Department of Urology, Cedars-Sinai Medical Center, Los Angeles, CA USA; 2 Veteran Affairs Health Care System, Durham, NC USA

**Keywords:** Prostate cancer, Health disparity, Race, Transcription factor, Network

## Abstract

**Background:**

Black men are at a higher risk of prostate cancer (PC) diagnosis and present with more high-grade PC than White men in an equal access setting. This study aimed to identify differential transcriptional regulation between Black and White men with PC.

**Methods:**

We performed microarray of radical prostatectomy tissue blocks from 305 Black and 238 White men treated at the Durham Veterans Affairs Medical Center. Differential expression, gene set enrichment analysis, master regulator analysis, and network modeling were conducted to compare gene expression by race. Findings were validated using external datasets that are available in the Gene Expression Omnibus (GEO) database. The first was a multi-institutional cohort of 1152 prostate cancer patients (596 Black, 556 White) with microarray data (GEO ID: GSE169038). The second was an Emory cohort of 106 patients (22 Black, 48 White, 36 men of unknown race) with RNA-seq data (GEO ID: GSE54460). Additionally, we analyzed androgen receptor (AR) chromatin binding profiles using paired AR ChIP-Seq datasets from Black and White men (GEO IDs: GSE18440 and GSE18441).

**Results:**

We identified 871 differentially expressed genes between Black and White men. White men had higher activity of MYC-related pathways, while Black men showed increased activity of inflammation, steroid hormone responses, and cancer progression-related pathways. We further identified the top 10 transcription factors (TFs) in Black patients, which formed a transcriptional regulatory network centered on the *AR*. The activities of this network and the pathways were significantly different in Black vs. White men across multiple cohorts and PC molecular subtypes.

**Conclusions:**

These findings suggest PC in Black and White men have distinct tumor transcriptional profiles. Furthermore, a highly interactive TF network centered on *AR* drives differential gene expression in Black men. Additional study is needed to understand the degree to which these differences in transcriptional regulatory elements contribute to PC health disparities.

**Supplementary Information:**

The online version contains supplementary material available at 10.1186/s13073-024-01361-0.

## Background

Black men in the United States exhibit one of the highest risks for prostate cancer in the world [1]. While much of these health disparities have been ascribed to socioeconomic differences and access to care, we previously showed Black (African-American) men are at higher risk of being diagnosed with PC, present more frequently with high-grade disease, are at increased risk of biochemical progression after radical prostatectomy (RP), and have higher expression of adverse molecular features, even in an equal access setting [2–4]. This strongly suggests that at least in part, biological differences between Black and White (European-American) men contribute to PC racial disparities (reviewed in Farrell et al. [5]), though the source of these distinctions, potentially including germline genetics, systemic racism, lifestyle factors, and socioeconomic differences, remains unclear.

Most genomic data in PC in the public domain is from White men (> 95%). However, in some studies, patterns of genomic aberrations suggest PCs in Black men are driven by oncogenic networks distinct from those in White men. For example, *ERG* translocations and/or *ERG* over-expression are seen in ~ 50% of White PCs but much less in Black tumors [6–8]. In a recent study of matched Black and White PCs, *ERG* was 3 times more likely to be overexpressed in high grade White PCs vs. Black PCs [7]. Given that it is now clear that PC can be divided into several subtypes [9, 10] and *ERG* over-expression defines a particular subtype of PC, this suggests the distribution of molecular subtypes varies by race. Indeed, we recently showed that across 5 previously described subtyping classification systems, the frequency of various subtypes varies by race [11]. However, no prior study of biological differences by race in PC has taken these differences in subtyping into account.

One approach to understanding biological underpinnings of PC racial disparities is to assess gene expression differences in tumors of Black and White men. A bioinformatic analysis of RNA expression data from 270 Black and 369 White PCs identified several pathways that predominated in one race vs. the other [7]. For example, inflammatory pathways were more commonly hyper-active in Black PCs. We [12] and others [13] have likewise noted higher expression of inflammatory pathway-related genes in Black PCs. Beyond inflammation-related gene expression, limited studies have examined other pathways that may differ by race. Our team identified differences in immune and DNA repair pathways by race [12], while another study noted differential expression of key PC biomarkers that appear to predict clinical outcomes in a race-specific manner [14]. However, to date, there has been no detailed analysis of racial differences with a focus beyond immune pathways nor has there been an attempt to identify key driver master regulator transcription factors. Moreover, no study to date has examined racial differences in gene expression accounting for known differences in PC subtypes. We hypothesized that a detailed bioinformatic analysis of a large cohort of PCs from Black and White men would identify multiple pathways differentially expressed by race, beyond immune pathways, and thereby provide unique insights into the distinct biology of PC in Black men. To accomplish this, we used a gene expression set of RP from a well-annotated patient cohort [15] and validated our findings using similar multiethnic cohorts.

## Methods

### Study cohorts

The Durham Veterans Affairs Health Care System (DVAHCS) cohort consisted of men treated with RP for clinically localized PC between 1989 and 2016 at the DVAHCS. Our inclusion criteria were pathologic stage pT2 with positive surgical margins, or pT3/4 disease, or received postoperative radiation for biochemical recurrence after RP. Biochemical recurrence was defined as one prostate-specific antigen (PSA) value above 0.2 ng/mL or two PSA values of 0.2 ng/mL after RP. Patients with positive lymph nodes at the time of RP were excluded. In addition, selected patients had to have available tumor tissue blocks and at least 2 post-RP PSA measurements. Patients with nodal or distant metastasis pre- or peri-operatively or who received any neo-adjuvant hormone or radiation therapy were excluded. Thus, our final sample size for gene expression analysis consisted of 543 subjects (305 Black and 238 White men) with complete data available for analysis. Race was abstracted from the electronic medical records. The DVAHCS Institutional Review Board reviewed and approved the research protocol under which this study was conducted using a waiver of informed consent. The research conformed to the principles of the Helsinki Declaration.

To compare gene expression by race, in conjunction with Decipher Biosciences, we performed Affymetrix Human Exon Array ST 1.0 assays of RP tissue blocks from 543 PC patients (305 Black and 238 White men) treated at the DVAHCS. The DVAHCS sees a high percent of Black men and is an equal access center, creating an ideal environment to study health disparities and biological differences by race. In this cohort, patient clinical and pathological characteristics were generally similar by race (Table 1), though Black men had higher PSA, were younger, and had more T2 tumors. RP tissue was reviewed by a pathologist to select the largest and highest-grade tumor for analysis. Tumor sampling was performed as previously described [15].

**Table 1:** Clinical characteristics of men undergoing radical prostatectomy in DVAHCS cohort

Clinical feature	Black	White	*P*-value
**Number of patients**	305	238	
**Age at surgery (years)**			
Mean	59.9	62.7	< 0.001^1^
Standard deviation	5.9	5.7	
**PSA prior to surgery (ng/ml)**			
Median	7.3	7.0	0.106^1^
Interquartile range	5.1, 11.4	5, 10.3	
**Pathological grade group, *n* (%)**			0.315^2^
1	30 (10%)	35 (15%)	
2	194 (63%)	137 (57%)	
3	48 (16%)	35 (15%)	
4	14 (5%)	11 (5%)	
5	18 (6%)	20 (8%)	
**Pathological stage, *n* (%)**			0.003^2^
T2	185 (61%)	116 (49%)	
T3	92 (30%)	105 (44%)	
T4	28 (9%)	17 (7%)	
**Positive surgical margins, *n* (%)**			0.614^2^
No	47 (15%)	33 (14%)	
Yes	258 (85%)	205 (86%)	
**Follow-up for men without recurrence (months)**			0.005^1^
Median	103.3	121.1	
Interquartile Range	64.9, 156.9	75.6, 170.6	
**Recurrent disease**			0.733^2^
No	148 (49%)	119 (50%)	
Yes	157 (51%)	119 (50%)	
**PCS subtypes**			
PCS1	5 (2%)	5 (2%)	0.698^2^
PCS2	170 (56%)	152 (63%)	0.067^2^
PCS4	131 (43%)	83 (35%)	0.051^2^

^1^*P*-values obtained from Wilcoxon rank-sum test

^2^*P*-values obtained from chi-square test

There are two independent PC cohorts included in this study for validation purpose. One is the multi-institutional retrospective study (MIRS) cohort of 1152 PC patients (596 Black and 556 White men) who underwent RP and had the Decipher whole-transcriptome assay [16]. The second is the Emory cohort, which consists of 106 samples (22 Black, 48 White, and 36 men of unknown race) that were taken from formalin-fixed, paraffin-embedded RP samples. Samples from the Emory cohort were obtained from the Atlanta VA Medical Center, U. Toronto Sunnybrook Research Centre, and Moffitt Cancer Center [17]. Both cohort datasets were obtained from Gene Expression Omnibus (GEO) database with accessions GSE169038 and GSE54460, respectively.

In this study, we used the terms White and Black (or African-American) consistent with the categories currently used by the United States Census Bureau (which collects data based on self-identification).

### Sample preparation and microarray data from the DVAHCS cohort

As previously described [15], central pathological review of formalin-fixed, paraffin-embedded RP tumor specimens from each eligible patient was conducted to re-grade tumors (according to International Society of Urological Pathology (ISUP) 2005 criteria). We selected tumor samples with > 60% tumor cellularity by area to minimize benign contamination from the region with the highest-grade group and, if present, extra prostatic extension or seminal vesicle invasion. Tumor was sampled using a 1.0-mm biopsy punch tool (Miltex, York, PA, USA). RNA extraction, cDNA amplification, and microarray hybridization were performed as previously described (Decipher Biosciences, Inc, San Diego, CA, USA) [18, 19]. Microarray quality control was applied using Affymetrix Power Tools packages [20], and probeset summarization and normalization were performed using the single-channel array normalization (SCAN) algorithm [21].

### Differential expression analysis of PCs from Black and White men

Genes with significant differential expression between Black and White men were identified using the integrated hypothesis testing method as previously reported. Briefly, (1) *T*-statistics, rank-sum statistics, and log2-median-ratio between the groups were computed for each gene. (2) Empirical distributions of the null hypothesis were estimated by calculating the three statistics for the genes after randomly permuting the samples 10,000 times. (3) For each gene, three *P*-values of the observed statistics were computed using their corresponding empirical distributions of null hypothesis by two-tailed test. (4) The three *P*-values were combined into an overall *P*-value using Stouffer’s method [22]. (5) Lastly, we performed multiple testing correction by using Storey’s method [23]. Genes with a false discovery rate (FDR) less than 0.05 and absolute log2-median-ratio greater than 0.0674 were defined as differentially expressed genes (DEGs). The cutoff value of log2-median-ratio was set at the 95-percentile value from the null distribution of log2-median-ratio, which is based on 1,000 random permutation of samples.

### Gene set enrichment analysis in each race

Gene-set enrichment analysis (GSEA) software was used for functional enrichment analysis [24]. Briefly, (1) the hallmark gene sets were obtained from Molecular Signatures Database (MSigDB) [25], (2) Log2-median-ratio between Black vs White men was used to order genes in a descending manner, (3) enrichment score (ES) was computed using a Kolmogorov–Smirnov running sum statistics for each gene set, and (4) significance of the ES was computed using a distribution of null hypothesis which was generated by doing 1000 random permutations.

### Master regulator analysis

TFs and their target interaction data were from our previous study [26]. The targets of individual TFs were counted among the DEGs. To compute the significance of number of TF target genes, we performed the following procedures: (1) the same number of genes as the number of DEGs were randomly sampled from the whole genome, (2) the target genes of each transcription factor were counted in the randomly sampled genes, (3) this procedure was repeated 100,000 times to generate an empirical null distribution, and (4) significance of an observed target count in the DEGs was computed using a one-tailed test with the empirical null distribution. Then, the Benjamini–Hochberg procedure was used to control the FDR [27].

### Network modeling of top 10 Black transcription factors

To generate a network model of top 10 TFs overactive in Black men compared to White men, (1) the proportion of number of targets in DEGs for each TF was calculated and mapped to size of node, (2) the activity difference of each TF was calculated and mapped to color of nodes, and (3) the proportion of co-regulated genes in DEGs were calculated and mapped to edge color and width.

### Analysis of AR chromatin binding profile

The AR chromatin immunoprecipitation sequencing (ChIP-Seq) data of Black and White men were downloaded from GEO database (GSE18440 and GSE18441). The Integrative Genomics Viewer (IGV) software was used to visualize AR binding signal. The plotHeatmap function in deepTools [28] was applied to obtain the signal around gene transcription start site (TSS) regions (up/down 2 kb) and generated the AR binding signal heatmap; the plotProfile function was applied to generate the line plot. The DESeq R package [29] was used to identify the differentially binding TSS regions of top TFs (10 and 3 TFs in Black and White men, respectively) with the cutoff |log2FC|> 1 and *P*-value < 0.01. As some candidate TFs have more than one TSS, the most significant *P*-value of a certain TF was displayed on the plots.

### Statistical analysis

The *Z*-score was used to calculate TF network activity [30]. The activity scores between Black and White men were compared using the Wilcoxon rank sum test. Fisher’s exact test was used to calculate the significance enrichment of pathways for each Prostate Cancer Classification System (PCS) categorization. The significance of overlap between DEGs from difference cohorts was calculated using chi-square test. The MATLAB package including the Statistics toolbox (Mathworks, Natick, MA, USA) was used for all statistical tests and computational analysis [31].

## Results

### Black and White PCs exhibit differential gene expression

**Fig. 1:**
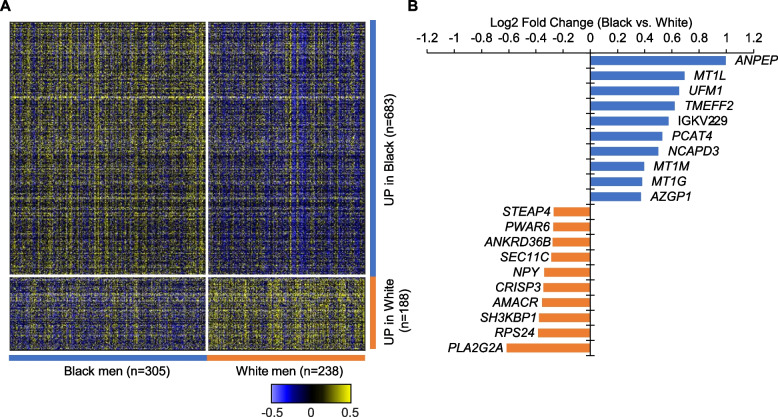
DEGs between Black and White men in the DVAHCS cohort. **A** Differential gene expression between Black and White men in the DVAHCS cohort. **B** Top 10 DEGs by race

We identified DEGs in PCs from Black compared to White men using the integrated hypothesis testing method. As a result, 683 genes were upregulated in Black men, and 188 genes were upregulated in White men (Fig. 1A). The top 10 DEGs for Black and White men are shown in Fig. 1B. *ANPEP* and *PCAT4* were in the top 10 DEGs in Black men, while *AMACR* and *NPY* were in the top 10 DEGs in White men. Of note, *AMACR*, whose overexpression is often used in clinical practice to confirm the diagnosis of PC on biopsy regardless of race, was overexpressed in tumors from White men relative to those from Black men [32].

### Differential enrichment of top pathways by race

**Fig. 2:**
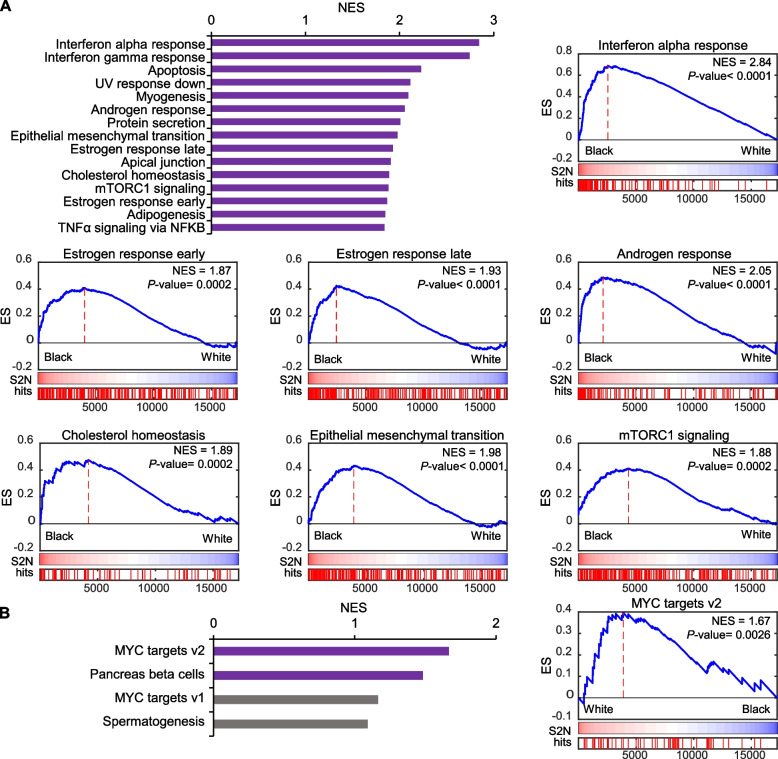
Enriched pathways in Black and White men in the DVAHCS cohort. **A** Top pathways enriched in Black men compared to White. **B** Top 4 pathways enriched in White men compared to Black. Purple bars represent significantly enriched pathways

Using the GSEA method [24], we identified the top pathways enriched in tumors from Black men (Fig. 2A) and the top pathways enriched in White men (Fig. 2B) with enrichment *P* < 0.05. In White men, there were only 2 significantly enriched pathways: MYC Targets v2 and pancreas beta cells. In contrast, Black men showed enrichment of many pathways, and thus, we limited analyses to the top 15 enriched pathways. These pathways included increased activity of inflammation-related pathways (interferon alpha response, interferon gamma response, mTORC1 signaling, and TNFα signaling via NFκB), steroid response-related pathways (cholesterol homeostasis and androgen and estrogen response), and cancer progression-related pathways (epithelial mesenchymal transition, apical junction, and adipogenesis).

### PC categorization reveals distinct enrichment of the pathways

**Fig. 3:**
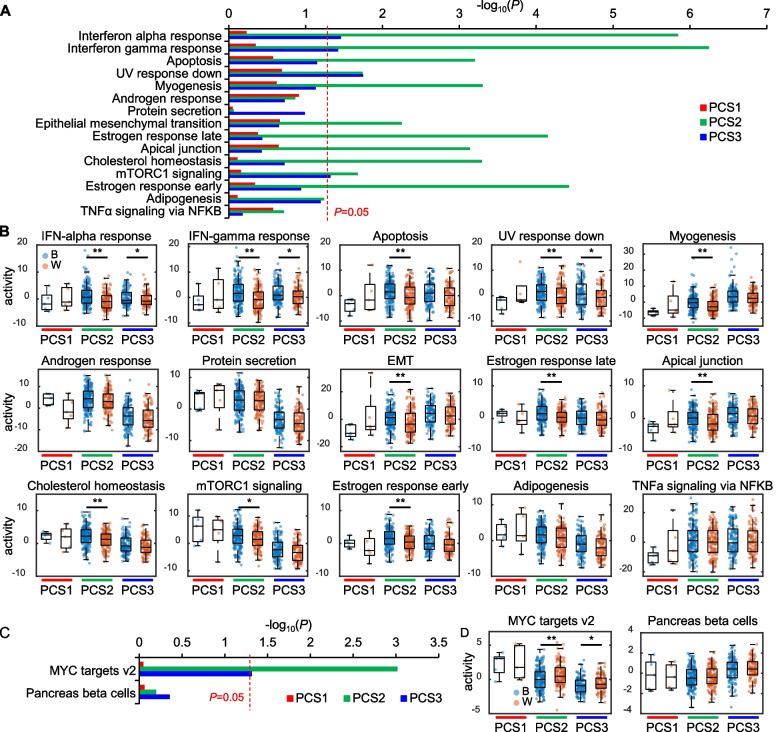
Differential enrichment of top pathways by PCS categorization. **A** Bar plot shows the significance of enrichment of pathways in Black men by PCS categorization. Red, green, and blue represent PCS1, 2, and 3, respectively. **B** Box plots show the activity of pathways enriched in Black men. **C** Bar plot shows significance of enrichment of pathways in White men by PCS categorization. **D** Box plot shows activity of pathways enriched in White men. Each dot represents the pathway activity of each patient. Blue dots represent Black men and orange dots represent White men

Given that Black and White men have different distributions of pathway enrichment patterns, we reanalyzed these data accounting for PC molecular subtype. Specifically, when tumors were stratified by PCS1 (5 Black and 5 White men; luminal subtype exhibited the poorest clinical outcomes), PCS2 (170 Black and 152 White men; luminal subtype with relatively favorable clinical outcomes), and PCS3 (131 Black and 83 White men; basal subtype with relatively favorable clinical outcomes) using the prostate cancer classification system (PCS) which are based on the activation signatures of 14 PCa-associated biological pathways [10], an interesting pattern emerged (Fig. 3A, B). Of the top 15 pathways enriched among all Black men, when stratified by PCS, 11 pathways related to inflammatory, sex steroid, and cancer progression were significant in PCS2 and 4 of them (inflammation-related pathways) were also significant in PCS3. No pathways were significant in PCS1 likely due to the small number of patients with the PCS1 subtype. Intriguingly, when each PCS was examined separately, androgen response, protein secretion, adipogenesis, and TNFα signaling via NFκB were not significantly different by race in *any* subtype, despite being significantly different by race when all samples were combined. For White men, enrichment of MYC Targets v2 was significantly enriched in both PCS2 and 3 subtypes, but not PCS1, likely again due to low patient numbers (Fig. 3C, D). In both White and Black men, the PCS2 subtype showed the greatest differences in pathway enrichment by race. This suggests that when stratified by PCS, racial differences are minimized in some subtypes vs. PCS2, where racial differences were most apparent. These data suggest that any attempt to analyze gene expression differences by race without accounting for molecular subtyping runs the risk of not only creating false positive associations but missing key data on which subtypes drive these differences.

### Validation of DVAHCS results in independent cohorts

**Fig. 4:**
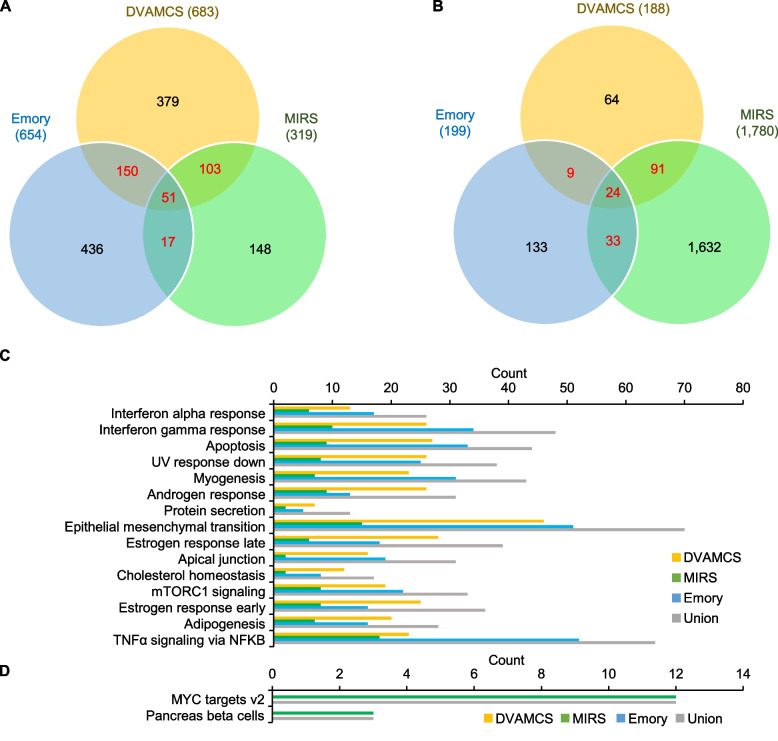
Common and distinct DEGs of Black and White men in three PC cohorts. **A**, **B** Venn diagram of DEGs of Black and White men from DVAHCS, MIRS, and Emory cohorts. **C**, **D** Number of DEGs from three PC cohorts that belong to top enriched pathways

To confirm whether the observed racial difference of gene expression was also present in independent cohorts, we identified two publicly available PC gene expression datasets from the MIRS (*n* = 1152; GSE169038) and Emory (*n* = 106; GSE54460) cohorts, respectively. We identified DEGs between Black and White men using an integrated hypothesis testing method [22] in the MIRS and Emory cohorts and compared them to DEGs in the DVAHCS cohort. Figure 4A and B depict the number of common and distinct DEGs between the three cohorts. In the Emory cohort, 654 genes were more highly expressed in Black men compared to White men, while 199 genes were more highly expressed in White men compared to Black men. In the MIRS cohort, on the other hand, 319 genes were more highly expressed in Black men compared to White men, while 1780 were more highly expressed in White men compared to Black men. Although the heterogeneity among cohorts is large, the number of overlapping genes among the three cohorts was significantly high. The DVAHCS and Emory cohorts had 201 (*P* < 0.0001) common DEGs in Black men and 33 (*P* < 0.0001) common DEGs in White men. The DVAHCS and MIRS cohorts had 154 (*P* < 0.0001) and 115 (*P* < 0.0001) common DEGs in Black and White men, respectively. From a functional point of view, upregulated genes in Black men from the MIRS and Emory cohorts were also involved in the top pathways enriched in Black men from the DVAHCS cohort (Fig. 4C). Highly expressed genes in White men from the MIRS cohort were also involved in the 2 pathways enriched in White men of the DVAHCS cohort. Although there are no overlapping genes involved in the 2 enriched pathways in White men from the Emory cohort (Fig. 4D), the GSEA result showed MYC target v2 was significantly enriched in White men (Additional file 1: Fig. S1; NES = 1.50 and *P* = 0.0104). The pancreas beta cells gene set was not significantly enriched in the Emory cohort.

### Different master regulators are active in PCs from Black and White men

**Fig. 5:**
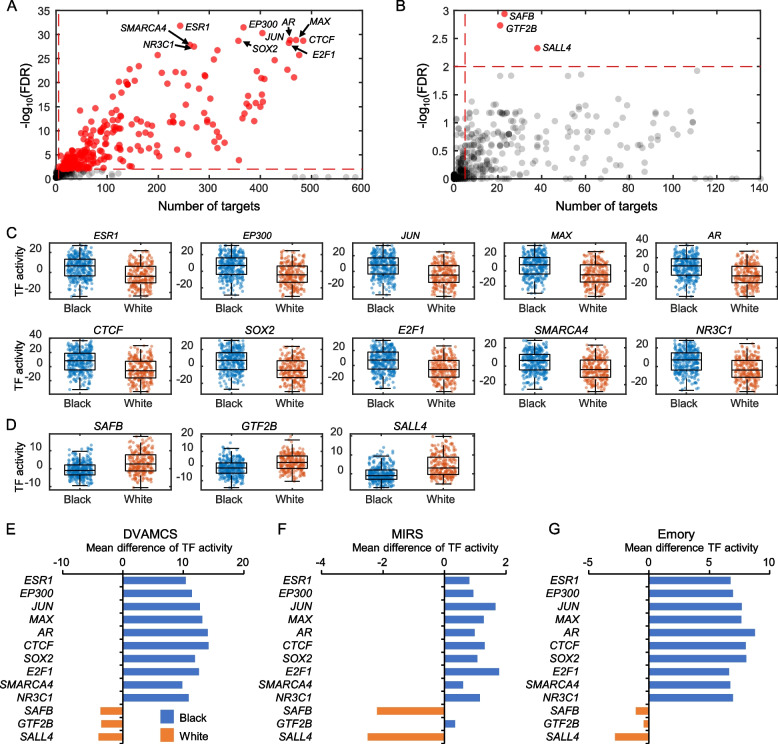
Key TFs in Black and White men. **A**, **B** Scatter plots show enrichment of TF targets in Black (**A**) and White (**B**) men. Red dots represent TFs that have enrichment FDR less than 0.01 and number of target genes larger than 5. **C**, **D** Box plots show distribution of activities of top TFs in Black (**C**) and White (**D**) men. **E**–**G** Bar plots show mean activity difference of top TFs from DVAHCS (**E**), MIRS (**F**), and Emory (**G**) cohorts

Given the differential gene expression and pathway activation by race, we hypothesized that distinct activation of transcriptional regulators may be driving these differences between Black and White men. We thus identified the top TFs active in PCs from Black and White men (Fig. 5A, B). Given many more TFs were overactive in Black men relative to White, we limited analyses to the top 10, while only 3 were specifically overactive in White men. A master regulator analysis was used to calculate the significance of number of potential target genes by using the collection of TFs and their target interactions. The criteria for being a master regulator (MR) included the following: (1) FDR less than 0.01, (2) the number of potential target genes larger than 5, and (3) top 10 TFs sorted by FDR. As a result, we identified 10 TFs in Black (*ESR1* [estrogen receptor-alpha], *EP300*, *JUN*, *MAX*, *AR*, *CTCF*, *SOX2*, *E2F1*, *SMARCA4*, and *NR3C1*) and 3 TFs in White men (*SAFB*, *GTF2B*, and *SALL4*). The activity of key TFs, the *Z*-score [30] of each TFs target genes, showed clear separation between tumors from Black and White men (Fig. 5C, D). These TF activity differences were also observed in the other two PC cohorts (Fig. 5E–G, Additional file 1: Fig. S2 and S3).

### A network of top 10 TFs has distinct activity between Black and White men

**Fig. 6:**
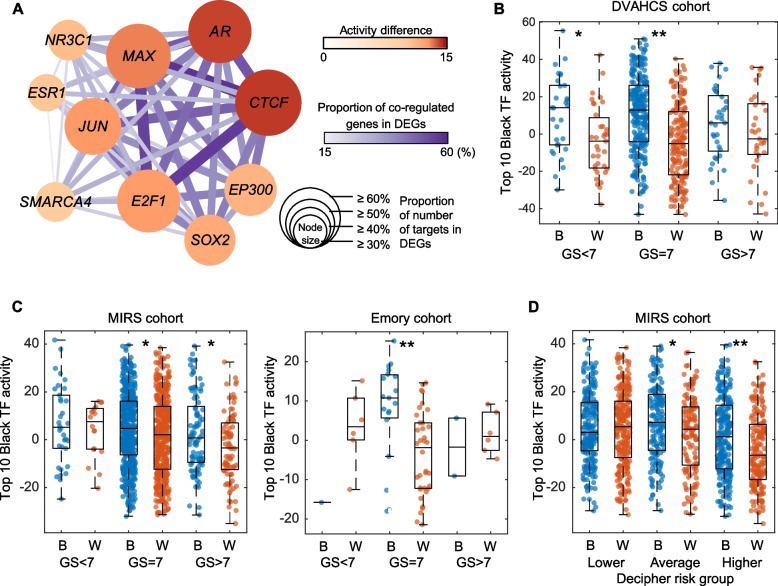
A network of top 10 TFs in Black men and its clinical association. **A** A network model shows the transcriptional regulatory network of the top 10 TFs in Black men. Node size represents the proportion of number of targets in DEGs. Node color represents the activity difference of 10 TFs between Black and White men in the DVAHCS cohort. Edge color and width represent the proportion of co-regulated genes in DEGs. **B** Box plot shows the distribution of top 10 Black TF activity by race and Gleason score in the DVAHCS cohort. **C** Box plot shows the distribution of top 10 Black TF activity by race and Gleason score in the MIRS (left) and Emory (right) cohorts. **D** Box plot shows the distribution of top 10 Black TF activity by race and Decipher risk groups in the MIRS cohort

To understand the transcriptional network that differentiates Black and White men, we reconstructed a network model using the top 10 TFs overactive in Black men compared to White men (Fig. 6A). Many target genes of the top 10 TFs, whose expression levels are significantly higher in Black men than White men, were co-regulated by these TFs (solid lines in Fig. 6A), suggesting that the highly interconnected TFs in the top 10 TFs network can regulate the same target genes simultaneously as a transcription complex. When patients were stratified by Gleason sum (GS), the top 10 TF network activity, which is the Z-score of union set of the 10 TFs target genes, showed a significant difference between Black and White men in all tumor grades, though this only reached significance in GS < 7 and GS = 7 (Fig. 6B; GS < 7, *P* = 0.0101; GS = 7, *P* < 0.0001). Consistently, the network activity in the MIRS cohort showed a significant difference between Black and White men with GS = 7 (Fig. 6C; GS = 7, *P* = 0.0168). The same was observed in the Emory cohort (Fig. 6C; GS = 7, *P* = 0.0011). When patients were stratified by genomic classifier scores, a prognostic measure strongly linked with clinical outcomes [18, 19, 33, 34], the higher the genomic classifier score, the greater overexpression of the enriched Black TF network in Black men relative to White men in MIRS cohort (Fig. 6D; average group: *P* = 0.0189; higher group: *P* = 0.0003). These results suggest that a distinct transcriptional regulatory framework operates in Black vs. White men, even in the same Gleason grade tumors, and that this regulatory network is even more differential by race in more aggressive tumors.

### Differential AR binding signal to the top TFs and their targets in Black and White men

**Fig. 7:**
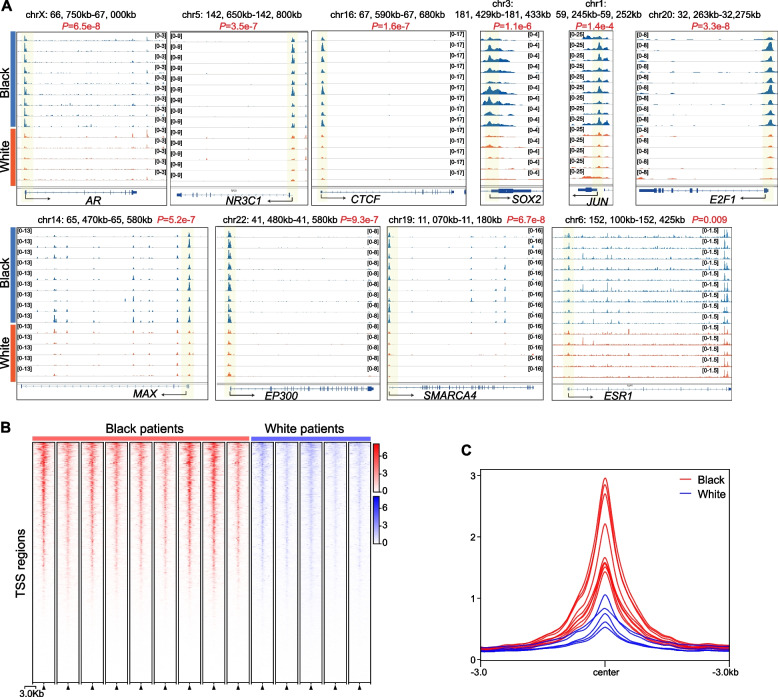
AR binding signal of the top 10 TFs in Black men in PC. **A** AR binding signal around top 10 TFs loci in Black men in published AR ChIP-Seq data. **B** Heatmap of AR binding signal in differentially expressed target gene at TSS regions of the top 10 TFs in Black and White patients. **C** Similar to **B**, line plots show the distribution of indicated AR ChIP-Seq signal at differentially expressed target gene TSS regions. The reference used here was hg19. TSS, transcription start site

To further validate whether top TFs have different AR binding patterns by race, we checked the AR binding signal using ChIP-seq (GSE18440 and GSE18441) [35]. All the top 10 TFs in Black men exhibited greater AR binding intensity around promoter regions of the top 10 TFs in Black men compared to White men (Fig. 7A). Notably, the TSS regions of all differentially expressed target genes from these TFs also showed higher AR binding intensity in Black men (Fig. 7B, C). These results revealed that AR can not only directly regulate top TFs but also regulate their target genes in Black compared to White PC patients. The AR binding intensity of the top 3 TFs (*SAFB*, *SALL4*, and *GTF2B*) and their differentially expressed target gene TSS regions in Black and White men are shown in Additional file 1: Fig. S4A-C. Of note, *SAFB* is an AR co-regulator, and loss of *SAFB* increased *AR* and PSA levels [36]. These findings suggest that *SAFB* drives a more aggressive phenotype in PC in Black men. *GTF2B* might also be the potential AR repressor gene in Black PC patients; however, this requires further exploration. Taken together, these results show that the top TFs and their target genes can be regulated by AR, consistent with the network connectivity pattern of MRs that are strongly connected around AR as shown in Fig. 6A.

## Discussion

Despite Black men having one of the highest risks and mortality rates of PC in the world, the biology of their tumors has hitherto been poorly studied. To overcome this, we examined detailed gene expression data from localized PCs from a large cohort of patients with a high representation of Black men (> 50%) within an equal access health system. A key finding of our study is the observation that many pathways differ by race. Consistent with multiple prior studies from our group and others [2–6, 12, 13], one of the biggest differences was higher activation of inflammatory pathways in tumors from Black men. However, we identified additional pathways not previously reported to be differentially activated by race, including increased activation of sex steroid pathways (androgen and estrogen), cholesterol homeostasis, and apoptosis in tumors from Black men. These results were validated in the MIRS and Emory cohorts where we observed a high overlap in genes involved in these pathways. We further identified key TFs in tumors from Black men that seem to help drive these differentially active pathways including the estrogen and cholesterol pathways. Finally, we identified a top 10 TF network, which differed by race and was highly active in Black men, especially among men with aggressive disease. Collectively, these results confirm that tumors from Black and White men have distinct sets of regulatory pathways and thus may offer unique opportunities for precision medicine for this population. We hope to build upon these findings to develop novel therapeutics targeting these differences in future studies to reduce PC health disparities.

The top two pathways significantly enriched in PCs of Black men compared to their White counterparts were inflammation related, as we hypothesized. Whereas Black men have higher incidence rates of PC and are more likely to die from PC, the reasons for this poor prognosis are complex and unclear. Chronic inflammation is known to predispose patients to developing different cancer types, including prostate carcinogenesis [37] and has been linked with PC progression [38]. Chronic inflammation can also induce both genetic and epigenetic alterations leading to microenvironmental changes that favor cell differentiation and tumorigenesis [39]. Therefore, it is not completely surprising to find enrichment of inflammatory pathways in Black men, who have higher PC risk. Besides serving as immunomodulators, interferons have been associated with resistance to cancer immunotherapy [40]. Type I interferons (e.g., IFN-α and IFN-β) serve as double-edged swords in cancer, promoting inflammatory responses, while initiating immunosuppressive feedback in both immune and cancer cells [41]. On the one hand, the immune promoting effects of IFNs (via stimulation of immune cells like macrophages and natural killers) enabled their use in the treatment of some cancers, while on the other hand, their sustained expression favors tumor progression. Based on an exploration of the PROCEED (NCT01306890) clinical data, where PC patients received ≥ 1 sipuleucel-T infusion, Black men had longer median overall survival compared to White men [42]. This suggests that inflammation patterns in PC from Black men, such as high interferon pathway activation, could lead to more favorable responses to immunotherapy in this patient population, though this requires future testing. In sum, the link between PC and inflammation is highly complex and should be studied within a specific context. As such, any differences in inflammation by race could play a role in PC health disparities. Future studies are needed to understand the temporal nature of the differences seen—especially as related to the immune system. More specifically, it is unknown whether the tumor altered the immune system or the immune system altered the tumor.

Beyond inflammation, the androgen and estrogen response pathways were significantly enriched in PC from Black men relative to those from White. At initial diagnosis, 80–90% of PCs are androgen-dependent, explaining why androgen-deprivation therapies are the first line of treatment [43]. AR signaling is also implicated in the development of castration-resistant prostate cancer (CRPC). While testosterone and 5α-dihydrotestosterone drive PC development and progression by binding to *AR*, *AR* itself can be stimulated by alternative molecules or pathways independent of androgens. Examples of signaling proteins capable of activating the *AR* include epidermal growth factor (*EGF*) [44], protein kinase A signal transduction pathway [45], and IL-6 through mitogen-activated protein kinase (MAPK) and STAT3 signal transduction pathways [46]. In addition, PCs are capable of synthesizing androgens de novo, and persistent intra-tumoral steroidogenesis is a suggested etiology of PC progression to CRPC [47]. The significant enrichment of the androgen response pathway in PC from Black men, relative to their White counterparts, may be explained by any of these mechanisms. Black men have higher estradiol levels than White men [48] and are exposed to higher levels of estrogen both during gestation and throughout their lives, and elevated levels of estrogen have been suggested to increase PC risk [49–51]. Whereas the relationship between estrogen levels and PC incidence and progression is the subject of ongoing research, our results suggest higher activity of the estrogen response pathway in PC from Black compared to White men. Whether this offers an opportunity for treatment/prevention remains unknown.

Similar to inflammation and steroid-related pathways, PC progression in Black men is associated with the upregulation of several molecular pathways. This includes the cholesterol homeostasis and adipogenesis pathways, which are linked to increased lipid metabolism and poor prognosis [5, 35, 52]. The epithelial-mesenchymal transition (EMT) pathway was also highly activated in tumors from Black men, with altered EMT processes in the tumor-adjacent stroma potentially responsible for the aggressive nature of PC in this population [53]. Enhanced EMT-related proteins, such as Snail, Vimentin, and Cathepsin L, were observed in PC cells from Black men compared to normal, androgen-dependent, and metastatic PC lines from White men [54]. Furthermore, the mTOR pathway was found to have higher activation in PC from Black men compared to White men, playing a critical role in disease aggressiveness and treatment response [55], as we also found the mTOR pathway was upregulated in Black men from the DVAHCS cohort. The crosstalk between the PI3K and mTOR pathways, with overexpression of key genes like PIK3CA, MTOR, and CD53 in Black versus White patients, is also thought to promote PC progression [56]. These findings suggest that therapeutic targeting of the top transcriptional regulators of these pathways could be a promising strategy to address racial disparities in PC outcomes.

There is limited information available on which TFs are active in PC from Black men, while a study found that the *AR* cistrome in PC in Black men is different from White men [35]. Master regulator analysis is a method used to infer the activity of TFs and identify MRs that control gene expression in each cell or tissue [57]. The advantage of this approach is that it can provide insights into the regulatory networks that underlie cellular processes and diseases. It can also help identify potential therapeutic targets for diseases that involve dysregulated gene expression. Here, we applied an MR analysis to transcriptome data from PC patients to identify if MRs differ by race. The resultant top TFs were identified and found to have critical function in PC biology and various interactions with *AR*. Recent studies revealed that *AR* is more active in Black PC patients compared to White, both at expression and epigenetic levels [35]. Furthermore, at a functional level, differential *AR* in Black men may contribute to higher levels of lipid metabolism, immune response, and cytokine signaling in PC [35]. Aside from *AR*, another top TF in Black men, *ESR1* (estrogen receptor alpha), is a sex steroid hormone receptor implicated in hormone-driven cancers, such as prostate and breast [58, 59]. *EP300*, *CTCF*, *SOX2*, and *E2F1* are activated in CRPC [60, 61]. *JUN* can interact with *AR* and function as a coactivator and repressor [62, 63]. *MAX* forms heterodimer with *MYC* and regulates PC growth [64]. *SMARCA4*, a member of SWI/SNF family, regulates *AR* activity by altering the chromatin structure [65]. *NR3C1* functions as a bypath of *AR* in androgen-targeted therapy resistant PC patients [66]. Among the top TFs in White men, *SAFB* is regulator of *AR* activity in PC [36]. These findings suggest TF activity and regulatory networks vary by race.

The significant enrichment of the androgen response pathway and the presence of AR in the top TFs in PC from Black men suggests that AR plays a crucial role in racial differences in gene expression. Using chromatin immunoprecipitation sequencing (Chip-seq) analysis, Berchuck et al. (2022) presented that differential binding of AR may contribute to distinct transcriptional programs [35]. In particular, the differential enrichment of Immune responses and lipid metabolic pathways between Black and White men are consistent with our GSEA results in Fig. 2. Analysis of the PC transcriptome produced by a microarray platform is a main feature of this study. A limitation of relying on gene expression data is that they may not always accurately reflect TF activity. In addition, our analysis assumes that TFs act independently, which may not always be the case. Finally, the identification of MRs may be influenced by the choice of algorithm and the quality of the input data [57]. To overcome this, we checked whether TFs showed a consistent pattern of activity in different cohorts and assessed *AR* chromatin binding profiles of the top TFs and their target genes. Although the two validation cohorts are independent cohorts with different clinical characteristics from the DVAHCS, and there is potential for bias due to platform differences, they still share a significant number of overlapping DEGs, similar functional enrichment patterns, and similar TF activities, giving an overall robustness to our findings. In addition to the methodological limitation, it is known that biological determinants of race in PC biology are not limited to transcriptional differences. Several studies show differential genomic and epigenomic alterations such as copy number alterations [67], genomic fusion of *TMPRSS2* and *ETS* TFs genes [68], and differential DNA methylation patterns [69] by race. Transcriptional differences can also result from epigenetic regulation, not just TF regulation. However, genomic data to assess African ancestry were not reliably available for this study. Moreover, our study was a bioinformatics analysis only without mechanistic verification. Further studies are needed to validate our findings at the mechanistic level. In addition, pathway activity analysis by PC molecular subtypes has less statistical power due to a small number of PCS1 tumors. Differential androgen regulation may further underlie relationships between patient race and PC molecular subtypes. Thus, further studies with large sample size would be helpful to determine if there are racial differences in advanced stages of the disease as well. Although this study provides the most active top 10 TFs in Black men, further research is needed to fully understand the active TFs related with the enriched pathways such as immune response. For instance, the transcription factor *NFκB* is highly expressed in PCs with high Gleason scores in Black men [70]. However, *NFκB* was not on the top 10 TFs in Black men. Lastly, other potential factors that could be driving racial differences in PC, such as lifestyle, associations with other diseases, and various social stressors such as structural racism, were not evaluated in this study. Consequently, we are unable to determine the root causes of these transcriptional differences. In sum, although we identified novel differences in transcriptional programming by race, additional research is needed to identify the key mechanisms by which we could reduce the biological impact of race in PC disparities.

## Conclusions

Transcriptome analysis of a racially diverse PC cohort allowed for the identification of key transcriptional regulators and molecular pathways significantly different in Black vs. White patients. However, the extent to which these differences in transcriptional regulatory elements contribute to PC health disparities will be the subject of further study.

### Supplementary Information

Additional file 1:  Fig. S1. GSEA result of MYC target v2 in the Emory cohort. Fig. S2. Activity of key TFs in Black and White men in the MIRS cohort. Fig. S3. Activity of key TFs in Black and White men in the Emory cohort. Fig. S4. AR binding signal of the top 3 TFs in White men and the expression correlation with AR in PC.

Additional file 2: Table S1. Clinical characteristics of men in Emory cohort. Table S2. Clinical characteristics of men in MIRS cohort.

## Data Availability

The transcriptome data of the DVAHCS cohort is available under controlled access from Decipher (http://decipher.sanger.ac.uk). The transcriptome data used for validation and the AR ChIP-Seq data were acquired from publicly available at Gene Expression Omnibus (GEO) (https://www.ncbi.nlm.nih.gov/geo/): GSE169038 (https://www.ncbi.nlm.nih.gov/geo/query/acc.cgi?acc=GSE169038) [16], GSE54460 (https://www.ncbi.nlm.nih.gov/geo/query/acc.cgi?acc=GSE54460) [17], GSE18440 (https://www.ncbi.nlm.nih.gov/geo/query/acc.cgi?acc=GSE18440) [71], and GSE18441 (https://www.ncbi.nlm.nih.gov/geo/query/acc.cgi?acc=GSE18441) [72].

## Abbreviations

PC: Prostate cancer
*AR*: Androgen receptor
TF: Transcription factor
RP: Radical prostatectomy
DVAHCS: Durham Veterans Affairs Health Care System
PSA: Prostate-specific antigen
MIRS: Multi-institutional retrospective study
GEO: Gene Expression Omnibus
ISUP: International Society of Urological Pathology
SCAN: Single-channel array normalization
FDR: False discovery rate
GSEA: Gene-set enrichment analysis
MSigDB: Molecular Signatures Database
ES: Enrichment score
DEGs: Differentially expressed genes
IGV: Integrative Genomics Viewer
TSS: Transcription start site
PCS: Prostate Cancer Classification System
MR: Master regulator
GS: Gleason sum
ChIP-Seq: Chromatin immunoprecipitation sequencing
CRPC: Castration-resistant prostate cancer
*EGF*: Epidermal growth factor
MAPK: Mitogen-activated protein kinase
EMT: Epithelial-mesenchymal transition
