# Supplementary material for: Prostate cancers with distinct transcriptional programs in Black and White men
Source: Genome Med. 2024 Jul 23;16:92. doi: 10.1186/s13073-024-01361-0 (PMC11267822; doi:10.1186/s13073-024-01361-0)
Supplement: Supplementary file 1 — Additional file 1: Fig. S1. GSEA result of MYC target v2 in the Emory cohort. Fig. S2. Activity of key TFs in Black and White men in the MIRS cohort. Fig. S3. Activity of key TFs in Black and White men in the Emory cohort. Fig. S4. AR binding signal of the top 3 TFs in White men and the expression correlation with AR in PC. [file 13073_2024_1361_MOESM1_ESM.pdf]

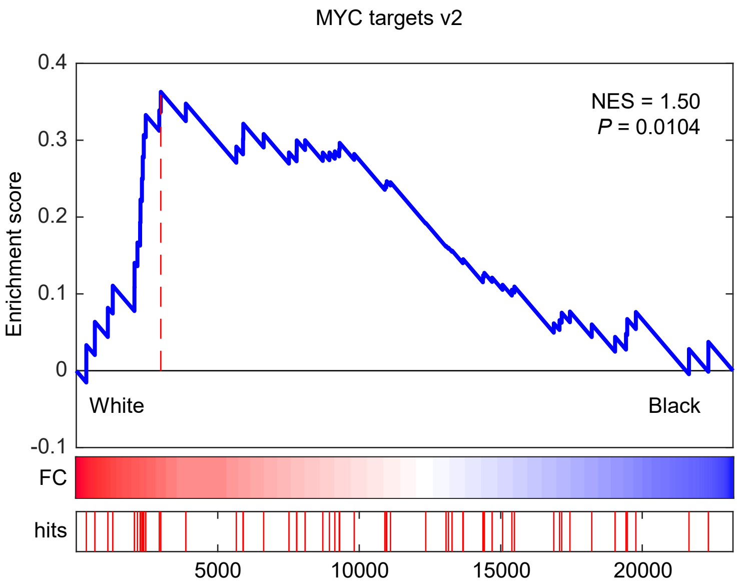

Fig S1. GSEA reslt of MYC target v2 in the Emory cohort.

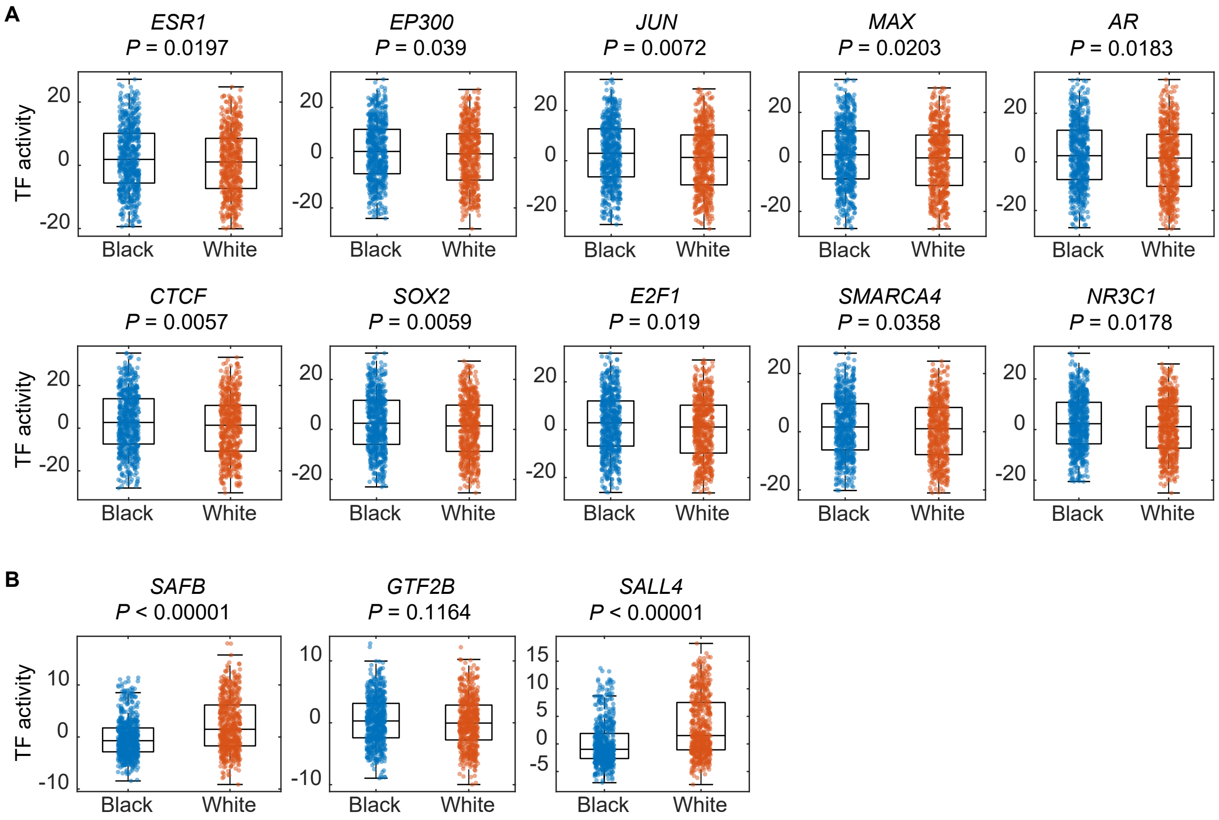

Fig S2. Activity of key TFs in Black and White men in the MIRS cohort. (A-B). Box plots show the distribution of activities of top TFs in Black (A) and White (B) men from the MIRS cohort.

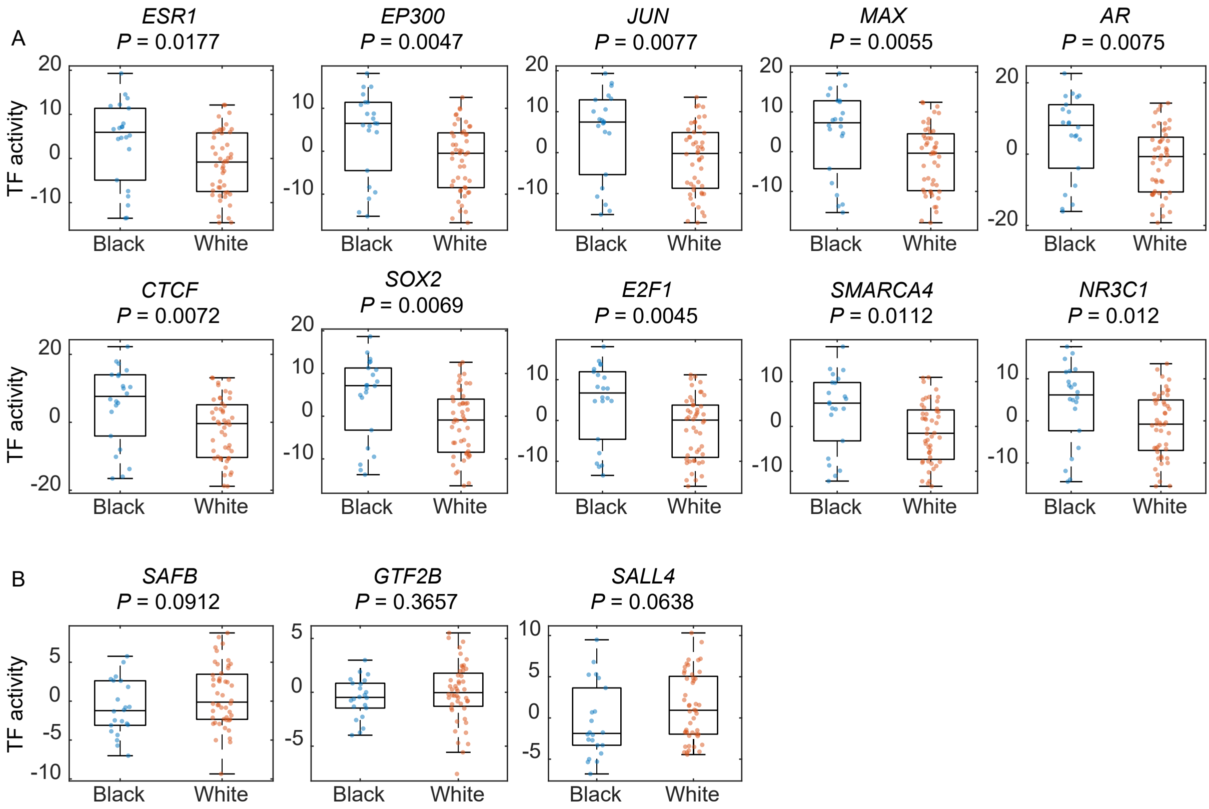

Fig S3. Activity of key TFs in Black and White men in the Emory cohort. (A-B). Box plots show the distribution of activities of top TFs in Black (A) and White (B) men from the Emory cohort.

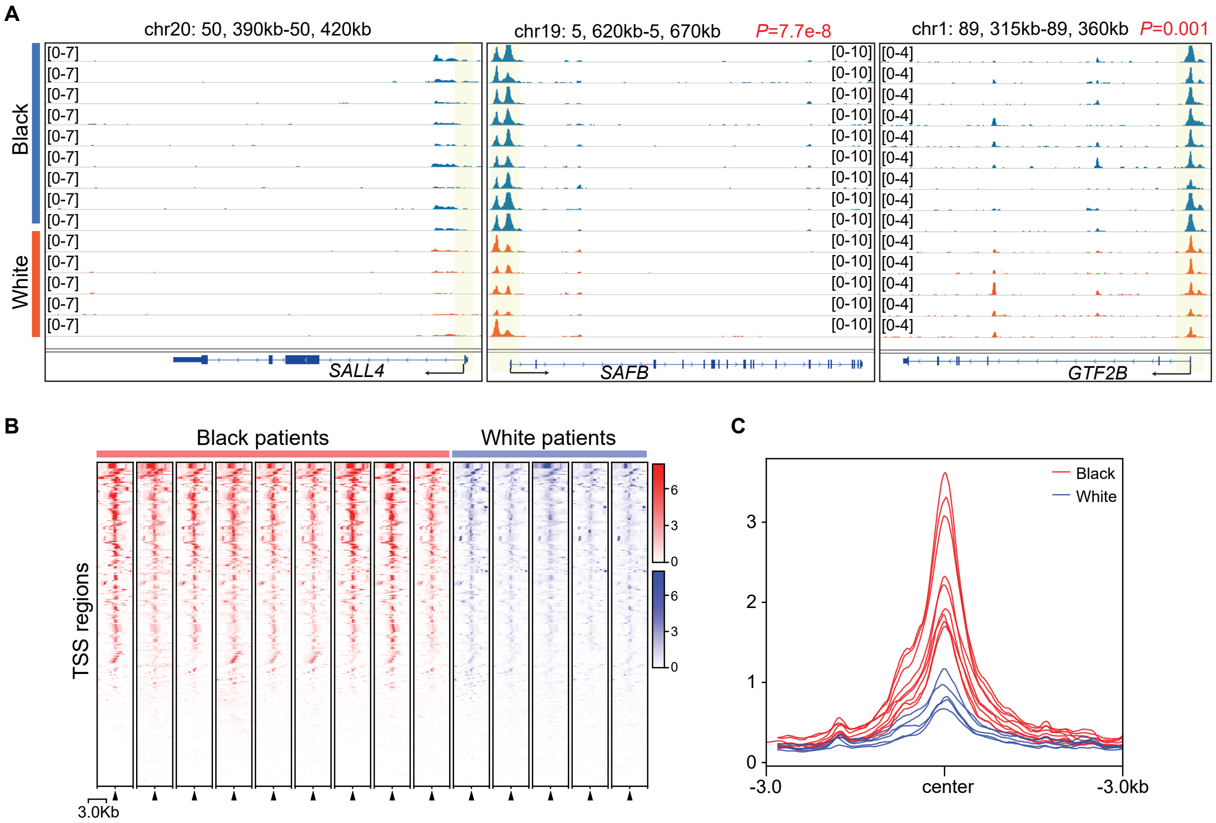

Fig S4. AR binding signal of the top 3 TFs in White men and the expression correlation with AR in PC. (A). AR binding signal around top 3 TFs loci in White men in published AR ChIP-Seq data. (B). Heatmap of AR binding signal in differentially expressed target gene TSS regions of the top 3 TFs loci in White men in published AR ChIP-Seq data. (C). Line plots show the distribution of indicated AR ChIP-Seq signal at differentially expressed target gene TSS regions.
